# Reevaluating the Impact of Epstein-Barr Virus Noncoding RNAs on the Interferon Response

**DOI:** 10.1128/mBio.00700-21

**Published:** 2021-08-24

**Authors:** Eain A. Murphy

**Affiliations:** a Microbiology and Immunology Department, SUNY Upstate Medical University, Syracuse, New York, USA

**Keywords:** EBV, interferon, miRNA, ncRNA, pDCs

## Abstract

The necessity of viruses to modulate the innate immune response often dictates the outcome of viral infection. As such, viruses encode many factors that undermine these potent antiviral responses. A recent study by Bouvet et al. (M. Bouvet, S. Voigt, T. Tagawa, M. Albanese, et al., mBio 12:e03440-20, 2021, https://doi.org/10.1128/mBio.03440-20) revisits the impact of virus-encoded noncoding RNAs on key components of the interferon pathway and sheds light on how the extensive biological functions of Epstein-Barr virus (EBV) microRNAs (miRNAs) are on targeting both the induction and signaling cascades of interferon.

## COMMENTARY

Each and every viral pathogen must undermine host antiviral responses in order to ensure a successful infection. The most potent and universal of these responses found within all nucleated cells is the induction of the type I interferon pathway (IFN-1) (an excellent review can be found in a book edited by Karen Mossman [1]). Activation of the IFN-1 pathway initiates a highly effective antiviral response in both the infected cells and neighboring uninfected cells. The activation of IFN-1 can be split into two separate phases. The first is the recognition of a foreign pathogen by the innate immune response mediators, the pattern recognition receptors (PRRs), which function as cellular sentinels monitoring for the presence of distinct motifs found within these pathogens. These motifs, called pathogen-associated molecular patterns (PAMPs) (2), are not present in uninfected cells; thus, IFN-1 activation is typically not induced in cells in the absence of virial infection. Upon recognition by PRRs, PAMPs induce a potent response culminating in the production of IFN-α and IFN-β, which represents the second phase of the IFN-1 response. Infected cells synthesize and secrete IFN-α and IFN-β, which can initiate natural killer cell functions and antigen presentation; however, their primary function is to bind to the interferon-α/β receptor (IFNAR), a nearly ubiquitously expressed membrane protein. Interferon secretion that results in binding to IFNAR on the same infected cell (autocrine activation) induces a signaling pathway that culminates in the transcriptional activation of interferon-stimulated genes (ISGs) that results in the limiting of viral replication, clearance of the pathogen, and initiation of antigen presentation. Neighboring noninfected cells also are activated by secreted IFN-α and IFN-β (paracrine activation) and thus render these cells inhospitable for the initiation of viral infection, thus limiting viral spread.

As IFN-1 activation is a potent inhibitor of viral replication, it is no surprise that viruses have coevolved with their hosts to undermine these responses. Viruses that lack the capacity to inhibit IFN-1 would be inefficient at establishing a productive infection and thus would eventually become extinct. Thus, successful viruses effectively undermine the IFN-1 pathway. In fact, each and every identified PRR, as well as each of the identified components of the IFNAR signaling pathway (3, 4), has been identified as a target of viral virulence factors, and the net effect of such targeting results in a dampening of the host antiviral response.

Viruses utilize multiple factors in their anti-interferon arsenal, including inhibitory proteins, molecular mimics and interferon decoys, proteins that bind to and inactivate the functions of the IFN-1 signaling pathway components, and factors that limit the levels of innate immune factors critical to the interferon pathway. It is becoming more apparent that viruses additionally exploit noncoding RNA species (ncRNAs), such as microRNAs (miRNAs), in the undermining of the host antiviral responses. miRNAs, short noncoding 22- to 23-nucleotide (nt) single-stranded RNAs first characterized over a quarter of a century ago (5), when loaded into the RNA inhibitory silencing complex (RISC), bind to a complementary seed sequence consisting of 6 to 8 nt within the 3′ untranslated region (UTR) of a target mRNA. Extensive binding within the miRNA:mRNA complex initiates the RISC endonuclease activity, which cleaves the double-stranded RNA (dsRNA) complex, thereby removing the poly(A) tail. This results in quick and efficient degradation of the mRNA. Less extensive binding inhibits the mRNA from being a suitable template for translation, resulting in reduced protein expression and eventual mRNA degradation.

At the time of the publication of this commentary, miRNA databases list more than 40,000 different miRNAs with a large proportion arising from viruses. This is not surprising as there are distinct advantages for viruses to encode miRNAs, including but not limited to the nonimmunogenic nature of these ncRNAs, as well as the nature of their small size (22 to 23 nt long), which is important for a pathogen with limited “genomic real estate” and allows them to be packaged within viral particles and exosomes. Thus, it is no wonder that viruses have evolved to exploit the use of miRNAs to alter host cell conditions that result in a favorable outcome for the virus. A master of exploiting miRNAs and noncoding RNAs to promote infection is Epstein-Barr virus (EBV) (reviewed in reference 6). EBV encodes 44 different miRNAs and two larger latently expressed EBV-encoded RNAs (EBERs), which are arguably the most studied of the viral encoded ncRNAs. These miRNAs and EBERs have various functions, many of which modulate the host immune response and cellular transformation (7). In this context, the miRNAs and EBERs can be viewed as EBV-encoded virulence factors. A recent publication by Bouvet et al. (8) reinvestigated the biological impact of encoded miRNAs and EBERs on the host antiviral response. With the addition of sequences found in clinical isolates of the virus that are lacking in laboratory bacterial artificial chromosome (BAC) strains, this study was able to generate a more-complete clinically relevant strain of the virus which was used to interrogate the requirement of the EBV miRNAs and EBERs. Building off this construct, the authors generated an array of highly engineered viral mutants lacking various ncRNAs or viral open reading frames (ORFs), all of which were previously reported to impact the interferon response upon infection (8). While generally there would be concerns that the simultaneous deletion of multiple viral factors might result in confounding results, the use of this physiologically relevant reagent allowed the authors to interrogate the requirement of these viral components in antiviral responses. To this end and in contrast to earlier studies that relied on established cell lines for analyses of the ncRNAs, this group elected to use primary cells, as innate and intrinsic immune responses in transformed cells likely fail to fully recapitulate the complex interferon responses observed in primary cells. The surprising findings from infection of the primary cells revealed that the principal viral regulators of diminished cytokine production were the EBV miRNAs, whereas the EBERs and the viral protein LF2, previously characterized as a type I interferon antagonist (9), were largely nonessential for inhibiting this antiviral response. Further, by using both *in silico* prediction analysis and an unbiased miRNA target detection methodology, argonaute-photoactivatable ribonucleoside-enhanced crosslinking and immunoprecipitation (AGO PAR-CLIP), the authors not only identified multiple, key interferon-mediating factors as direct targets of the viral encoded miRNAs but found that ISG-driven transcription is negatively impacted in the presence of the miRNAs (8). Underscoring the effectiveness of undermining the interferon response, EBV employs redundancy in targeting interferon pathways by inhibiting several host factors which function at distinct stages of the same pathway. Using a highly comprehensive evaluation involving both *in silico* and PAR-CLIP analyses, coupled with target 3′ UTR reporter constructs, allowed for a detailed empirical characterization of miRNA targeting. The authors provide compelling evidence that miRNAs encoded by EBV target key type I interferon inducers (e.g., RIG-1, IRAK2, and OAS2). As described above, there are two axes involved in the IFN-1 response: (i) pathogen detection inducing IFN production and (ii) the downstream signaling induced by IFN binding to its cognate receptor. Bouvet et al. (8) found that EBV effectively inhibits both arms of the IFN response by targeting multiple factors in both pathogen recognition and IFN production, as well as mediators of the signal transduction cascade, whereas EBERs and LF2 had negligible effects on either arm in primary cells. This is a surprising finding, as previous studies have implicated the EBERs and LF2 as potent regulators of the interferon response (9, 10). What differs in this study is the characterization of the biological functions of these viral factors within primary cells as opposed to well-established cell lines in which the innate immune response pathway is most likely already dysregulated due to the transformation process.

At the risk of being overly simplistic, viral infection initiates similar changes as what one observes during transformation. Essentially, upon infection, a virus converts an otherwise normal cell into a “factory” to make more virus in a fashion similar to how a transformed cell is converted into a “factory” to make more transformed cells. This conversion involves similar changes in the microcellular environment, such as alterations in metabolic activity, DNA damage responses, fatty acid generation, and induction of the Warburg effect, to name a few. This is why researchers often depend on transformed cell lines for propagation of difficult-to-grow viruses; the work is already halfway done for the virus in transformed cells. Importantly, and relevant for this study, this conducive environment includes alterations to the innate immune pathways. Thus, it is not overly surprising that one would observe different, and possibly opposite, effects of viral immune modulators in the context of infection of primary versus transformed cell lines, as Bouvet and colleagues observed in this study.

Beyond the profiling of distinct IFN inhibition within primary cells, Bouvet et al. further identified the source of IFN-α production in response to EBV infection. It is well established that the primary site of infection by EBV is B cells within the peripheral blood. B cells are both susceptible and permissive for infection and are the lineage of cells that can be transformed during latent infection with the virus. While B cells have the capacity to generate significant amounts of IFN-α (11), the characterization of the cell type involved in the production of this cytokine within EBV-infected peripheral blood mononuclear cells (PBMCs) remained incomplete. The authors of this current study developed a novel viral fusion monitoring assay that allows one to identify cell types that bind and fuse with a reporter EBV, thereby allowing assessment of cell characteristics in a time frame that does not require *de novo* viral or host protein synthesis. The authors report that the predominant source of IFN-α is plasmacytoid dendritic cells (pDCs), a specialized subset of dendritic cells whose function is to detect pathogens and mount a robust interferon response (12), and not the resident B cells in the infected PBMCs. pDCs are susceptible but not permissive for EBV infection; thus, they would inherently be missed by standard reporter virus infection characterization. It should be noted that IFN-α production in the context of other viral infections is driven by pDCs, including Middle East respiratory syndrome coronavirus (13), dengue virus (14), tick-borne encephalitis virus (12), and the current pandemic pathogen SARS-CoV-2 virus (15), to name a few. While Bouvet et al. show the pDCs are the predominant source of EBV-induced IFN-α release, the actual total percentage of pDCs that are responsive to EBV is relatively small (∼5 to 7%). Importantly, this percentage did not rise with increasing viral load, thereby suggesting there are distinct populations of pDCs that are responsive to EBV, though further studies are necessary for subcharacterization of these important cell types.

The surprising findings of Bouvet et al. are that EBV infection targets the interferon response so systematically. Whereas one would imagine that inhibition of a couple of key factors would render the pathway nonfunctional, EBV ncRNAs, specifically the miRNAs, target nearly every substep of IFN activation, thereby ensuring a productive infection. The coevolution of EBV with its host, humans, suggests that this comprehensive undermining of the innate immune response has allowed EBV to establish infections in a majority of the human population (up to 90% in individuals over the age of 20 years [16]). It should be noted that these studies go beyond identifying the distinct targets of viral encoded ncRNAs impacting innate immune responses. This work highlights the importance of using physiologically relevant factors (reconstituted complete viral strains, primary cells, etc.) coupled with novel approaches (viral fusion marked cell identification) not only to accurately and mechanistically define previously overlooked virus-host interactions but also to identify the relevant cell types critical for antiviral responses.
